# Supplementary material for: Fitness Cost of Antiretroviral Drug Resistance Mutations on the pol Gene during Analytical Antiretroviral Treatment Interruption among Individuals Experiencing Virological Failure
Source: Pathogens. 2021 Nov 3;10(11):1425. doi: 10.3390/pathogens10111425 (PMC8622617; doi:10.3390/pathogens10111425)
Supplement: Supplementary file 1 [file pathogens-10-01425-s001.zip › pathogens-1266514-supple/S1_table_pol.pdf]

Class	*Drug*	*Number n = 36*	*Percent of Cases*
NRTI	abacavir (ABC)	3	8.33
NRTI	AZT/3TC	4	11.11
NRTI	didanosine (ddI)	25	69.44
NRTI	lamivudine (3TC)	31	86.11
NRTI	stavudine (d4T)	30	83.33
NRTI	zidovudine (AZT)	31	86.11
NNRTI	delavirdine (DDC)	4	11.11
NNRTI	efavirenz (EFV)	3	8.33
NNRTI	nevirapine (NVP)	15	41.67
PI	indinavir (IDV)	26	72.22
PI	saquinavir (SQV)	23	63.89
PI	ritonavir (RTV)	29	80.56
PI	nelfinavir (NFV)	12	33.33
PI	amprenavir (APV)	2	5.56
