# Supplementary material for: Fitness Cost of Antiretroviral Drug Resistance Mutations on the pol Gene during Analytical Antiretroviral Treatment Interruption among Individuals Experiencing Virological Failure
Source: Pathogens. 2021 Nov 3;10(11):1425. doi: 10.3390/pathogens10111425 (PMC8622617; doi:10.3390/pathogens10111425)
Supplement: Supplementary file 1 [file pathogens-10-01425-s001.zip › pathogens-1266514-supple/S2B_Group_Data.pdf]

			Reversion Group		
			**None**	**Low**	**High**
**Viral Load**	Baseline	Mean	5.11	5.08	4.76
		Std. Dev.	0.73	0.35	0.53
	Week 4	Mean	5.22	5.58	5.29
		Std. Dev.	0.61	0.27	0.61
	Week 8	Mean	5.39	5.71	5.34
		Std. Dev.	0.61	0.37	0.62
	Week 12	Mean	5.25	5.91	5.51
		Std. Dev.	0.62	0.46	0.51
**CD4+**	Baseline	Mean	244.08	229.00	266.25
		Std. Dev.	185.35	173.80	113.11
	Week 4	Mean	224.62	193.93	207.63
		Std. Dev.	187.60	146.06	72.25
	Week 8	Mean	209.15	145.60	196.13
		Std. Dev.	179.10	123.46	104.41
	Week 12	Mean	163.54	120.53	155.13
		Std. Dev.	142.90	131.91	110.57
**CD8+**	Baseline	Mean	806.92	866.47	1168.25
		Std. Dev.	480.38	426.25	943.21
	Week 4	Mean	808.85	970.73	1057.63
		Std. Dev.	552.45	449.64	516.69
	Week 8	Mean	924.00	848.33	927.63
		Std. Dev.	494.44	409.64	563.85
	Week 12	Mean	733.62	785.00	785.88
		Std. Dev.	431.92	472.76	356.40
