# Supplementary material for: Fitness Cost of Antiretroviral Drug Resistance Mutations on the pol Gene during Analytical Antiretroviral Treatment Interruption among Individuals Experiencing Virological Failure
Source: Pathogens. 2021 Nov 3;10(11):1425. doi: 10.3390/pathogens10111425 (PMC8622617; doi:10.3390/pathogens10111425)
Supplement: Supplementary file 1 [file pathogens-10-01425-s001.zip › pathogens-1266514-supple/S2_table_pol_pacdata.pdf]

Patient No.	Viral Load				CD4+				CD8+			
	Baseline	Week 4	Week 8	Week 12	Baseline	Week 4	Week 8	Week 12	Baseline	Week 4	Week 8	Week 12
1	130,000	260,000	480,000	400,000	615	505	425	503	1,668	1,174	1,008	1,470
2	17,000	450,000	200,000	350,000	590	213	201	253	1,414	862	714	675
3	290,000	350,000	230,000	320,000	468	301	345	205	2,791	1,699	2,001	1,197
4	290,000	270,000	280,000	360,000	409	289	239	187	1,362	1,860	1,372	1,130
5	98,000	150,000	290,000	710,000	218	163	124	81	630	931	875	596
6	48,000	49,000	49,000	21,000	422	313	439	330	1,050	630	1,129	867
7	22,000	6,800	18,000	100,000	444	439	394	345	1,012	1,122	870	802
8	610,000	290,000	380,000	420,000	105	84	61	65	763	929	906	873
9	140,000	290,000	160,000	540,000	267	190	204	142	1,216	1,274	1,199	954
10	190,000	120,000	180,000	170,000	27	NA	NA	NA	381	412	363	380
11	1,700,000	3,100,000	2,400,000	2,400,000	20	NA	NA	NA	153	121	137	112
12	490,000	210,000	170,000	110,000	278	260	269	267	683	926	970	982
14	1,000,000	390,000	1,200,000	1,000,000	30	24	19	11	198	289	312	248
15	540,000	390,000	1,200,000	420,000	65	48	72	46	192	171	1,084	480
19	540,000	730,000	1,900,000	1,300,000	289	113	80	16	850	279	292	134
21	18,000	14,000	22,000	27,000	385	289	315	368	1,468	1,647	1,562	1,438
22	280,000	550,000	300,000	830,000	210	195	156	102	561	678	624	534
23	700,000	2,100,000	1,400,000	1,100,000	10	10	13	12	364	358	618	460
24	64,000	630,000	1,100,000	300,000	271	154	135	93	2,375	1,618	870	859
26	230,000	510,000	970,000	990,000	506	402	265	230	1,333	1,609	1,453	1,386
27	320,000	1,700,000	2,000,000	1,500,000	117	137	66	35	498	751	409	484
28	810,000	1,100,000	2,700,000	7,800,000	62	114	41	25	296	586	683	390
29	54,000	150,000	170,000	47,000	543	463	437	357	1,413	1,636	1,657	1,509
34	17,000	160,000	170,000	170,000	287	336	323	195	1,486	1,891	1,267	1,199
35	40,000	100,000	190,000	160,000	367	414	317	251	1,183	1,329	1,582	1,194
36	95,000	670,000	2,100,000	4,300,000	65	56	30	20	647	562	526	320
37	26,000	93,000	120,000	130,000	459	529	440	293	1,586	1,322	1,897	1,275
41	150,000	450,000	650,000	810,000	183	110	72	24	914	1,052	1,051	661
43	36,000	170,000	160,000	290,000	165	186	175	103	510	930	733	505
44	43,000	100,000	71,000	480,000	169	121	102	67	674	770	619	446
46	91,000	210,000	760,000	2,200,000	212	163	86	78	954	1,144	603	1,390
47	82,000	400,000	660,000	320,000	45	28	21	10	414	311	325	169
48	9,400	61,000	100,000	16,000	214	300	169	188	512	550	647	328
50	77,000	460,000	640,000	1,700,000	173	126	92	86	983	1,092	795	1,157
51	13,000	140,000	150,000	180,000	241	231	266	234	301	399	498	620
52	79,000	890,000	650,000	620,000	74	30	16	14	692	481	489	342
53	66,000	450,000	350,000	700,000	82	101	45	33	264	479	282	173
56	21,000	190,000	160,000	460,000	261	265	216	158	609	646	587	647

Table S2. Viral Load, CD4 and CD8 Counts for all patients at all 4 test points. All patients are subtype B except 3 and 53 who are B/F.
