# Supplementary material for: Fitness Cost of Antiretroviral Drug Resistance Mutations on the pol Gene during Analytical Antiretroviral Treatment Interruption among Individuals Experiencing Virological Failure
Source: Pathogens. 2021 Nov 3;10(11):1425. doi: 10.3390/pathogens10111425 (PMC8622617; doi:10.3390/pathogens10111425)
Supplement: Supplementary file 1 [file pathogens-10-01425-s001.zip › pathogens-1266514-supple/S3_table_pol_mutations_drugs.pdf]

Mutations per Patient -- pre and post ATI

	tr41		tr67		tr69		tr70		tr74		tr75		tr103		tr118		tr179		tr181		tr184		tr188		tr190		tr208		tr210		tr211		tr214		tr215		tr219		pr10		pr20		pr24		pr30		pr36		pr46		pr48		pr53		pr54		pr63		pr71		pr73		pr77		pr82		pr84		pr88		pr89		pr90		pr93		Drugs Used Pre-ATI	
	pre	post	pre	post	pre	post	pre	post	pre	post	pre	post	pre	post	pre	post	pre	post	pre	post	pre	post	pre	post	pre	post	pre	post	pre	post	pre	post	pre	post	pre	post	pre	post	pre	post	pre	post	pre	post	pre	post	pre	post	pre	post	pre	post	pre	post	pre	post	pre	post	pre	post	pre	post	pre	post	pre	post	pre	post	pre	post	pre	post	pre	post	PR Inhibitors	RT Inhibitors
pac1	x	x						x					x	x			x		x	x	x	x								x	x	x	x	x	x	x			x	x		x					x	x	x	x					x	x	x	x	x	x						x	x	x									IDV, SQV, RTV+SQV	AZT, DDC, ddI, 3TC+d4T
pac3	x	x	x	x		x	x												x	x	x	x			x						x	x	x		x	x				x		x	x				x	x				x	x	x	x	x	x						x	x		x					x	x					IDV, RTV+SQV	AZT+ddI, AZT+3TC, d4T+NVP
pac4			x	x														x	x	x	x	x											x	x	x	x	x		x		x	x					x	x			x	x			x	x		x							x	x				x						x	IDV, RTV+SQV, IDV+NFV	AZT+ddI, d4T+3TC, NVP
pac5	x	x		x			x	x											x	x	x							x			x	x	x	x	x	x	x	x			x	x						x	x								x	x		x	x	x						x						x				
pac6													x	x						x	x	x				x					x	x	x	x	x	x					x	x					x	x									x	x	x	x		x											x	x			IDV, SQV, RTV+SQV, SQV+NFV	AZT+3TC, d4T+3TC, d4T+ddI
pac7																																																																													APV	ABC+EFV
pac8	x	x	x	x			x	x					x						x	x							x						x		x	x		x		x									x	x				x									x	x			x	x	x	x							IDV, RTV+SQV	AZT+ddI, d4T+3TC, d4T, NVP
pac9			x	x			x	x										x	x	x	x																x					x					x	x								x	x	x	x	x	x	x					x	x		x			x	x			IDV, RTV+SQV	d4T+3TC, AZT+3TC
pac10			x	x	x	x	x	x								x				x	x	x	x	x											x	x	x	x		x							x	x	x	x							x	x		x		x				x	x	x					x	x		x		AZT+3TC
pac12	x	x			x	x							x	x																				x						x			x	x					x	x							x	x					x	x	x	x											IDV, RTV+SQV	AZT+ddI, AZT+3TC, d4T+NVP
pac14	x	x											x	x							x	x									x	x	x	x	x	x					x	x						x	x	x							x	x	x	x													x	x			IDV, RTV+IDV, RTV+SQV, NFV	AZT+ddI, AZT+3TC, d4T+NVP
pac15	x	x	x	x	x	x						x	x	x					x	x	x	x							x	x	x	x		x	x	x				x						x	x	x	x	x						x	x	x							x					x						x	IDV, NFV, RTV+IDV	AZT+ddI, AZT+3TC, d4T+NVP
pac19	x	x	x	x												x			x	x	x	x			x	x			x	x			x	x	x	x	x	x		x		x					x	x							x	x	x	x	x	x					x	x							x	x				
pac21	x	x												x					x	x	x	x				x					x	x	x	x	x	x				x								x	x	x					x	x	x	x		x			x	x		x							x	x		x		
pac22			x	x			x	x											x	x	x	x											x	x	x	x	x			x	x	x						x		x							x	x	x	x							x	x					x	x			IDV, RTV+SQV	AZT+ddI, d4T+3TC, ddI+ABC+EFV
pac23			x	x															x	x									x	x			x	x	x	x	x	x					x	x	x	x	x		x	x							x	x	x	x			x	x	x	x	x	x								x	IDV, RTV+SQV, RTV+NFV	AZT+ddI, d4T+3TC, NVP
pac24		x											x	x							x	x								x		x	x	x	x	x		x		x		x		x						x						x	x	x	x	x					x	x	x	x									RTV, SQV+NFV	AZT+ddI, AZT+ddI+NVP, d4T+3TC
pac26	x	x																	x	x	x	x									x	x	x	x	x	x			x										x	x							x	x	x	x					x		x	x									IDV, RTV+IDV	AZT+ddI, AZT+3TC, d4T+NVP
pac27	x		x	x			x	x													x	x			x	x							x	x	x	x	x	x	x	x	x	x					x	x							x	x	x	x							x	x											RTV+IDV	d4T+3TC
pac28	x	x	x	x					x										x	x	x	x						x		x		x		x	x	x	x			x							x	x	x	x							x	x	x	x		x	x	x			x	x					x	x			IDV, RTV+SQV, RTV+NFV	AZT+ddI, d4T+3TC, d4T+ NVP, AZT+ddI
pac29	x	x	x	x			x	x													x	x			x	x					x	x		x	x	x	x	x			x	x					x	x	x	x					x	x	x	x	x	x				x													IDV, RTV+IDV	AZT+3TC, d4T+3TC
pac34	x	x	x				x						x	x							x	x									x	x	x	x	x	x																				x	x	x																	x	x	x	x	IDV, RTV+SQV	AZT+ddI, AZT+3TC, d4T+3TC, d4T+NVP
pac35			x	x			x	x					x	x						x												x		x						x		x					x	x			x	x			x	x	x	x						x													RTV, RTV+SQV, NFV+SQV	AZT+ddI, d4T+3TC, d4+ NVP
pac36	x	x														x					x	x			x	x				x			x	x	x	x				x								x	x	x					x	x	x	x	x	x					x	x		x		x			x	x			IDV, RTV+SQV	AZT+ddI, d4T+ddI
pac37																																																																x													IDV, RTV+IDV, SQV+NFV	AZT+ddI, d4T+3TC, d4T+ddI
pac41	x	x		x				x					x	x					x	x		x			x	x			x	x	x	x	x	x	x	x	x		x		x	x					x	x					x		x	x	x	x	x	x					x	x							x	x			RTV, SQV+NFV	AZT+ddI, 3TC+d4T, d4T+NVP
pac43	x	x								x			x	x				x	x	x	x	x										x	x	x	x	x												x								x	x	x	x	x													x	x		x	IDV, RTV+SQV	AZT+ddI, 3TC+d4T, d4T+NVP
pac44	x	x	x																x	x	x	x			x	x	x		x	x	x	x	x	x	x	x	x	x												x							x	x	x	x		x		x			x	x		x			x	x	x	x	IDV, RTV+SQV	AZT+d4T, d4T+3TC, D4T+DDC, AZT+3TC
pac46	x	x	x	x					x	x	x		x	x		x			x	x					x					x		x	x	x	x	x				x	x	x					x	x		x	x	x			x	x	x	x	x	x					x	x				x			x	x	x	x	IDV, RTV+IDV, SQV+NFV	AZT+ddI, d4T+3TC, d4T+3TC+DLV, d4T+ddI+DLV
pac47	x	x	x	x			x	x	x							x			x	x	x	x		x						x		x	x	x	x	x	x	x		x							x	x	x	x				x	x	x	x	x	x	x							x	x					x	x	x	x	IDV, RTV+SQV	AZT+ddI, 3TC+d4T, ddI+ABC+EFV
pac48	x	x	x	x				x											x	x	x	x									x	x	x	x	x		x	x									x	x							x	x	x	x									x	x					x	x			IDV, RTV+IDV	AZT+ddI, AZT+3TC, d4T+NVP
pac50	x	x	x							x			x	x					x		x	x			x				x	x			x	x	x	x			x										x	x						x	x	x	x	x	x	x	x	x			x	x					x	x		x	APV	ABC+EFV
pac51	x	x											x	x							x							x				x	x		x	x	x	x		x								x							x	x	x	x		x				x		x							x	x	x	x	IDV, SQV, RTV+SQV, SQV+NFV	AZT+3TC, d4T+3TC, d4T+ddI
pac52			x	x			x	x													x	x			x	x					x	x	x	x	x	x	x	x	x	x			x	x							x	x		x	x	x									x	x	x	x									NFV, RTV	AZT+ddI, d4T+3TC, ABC
pac53							x	x					x	x						x	x	x		x	x			x					x	x	x	x	x	x			x	x														x					x	x	x	x									x	x			SQV, NFV	AZT+ddI, d4T+3TC, DDC NVP+DLV+EFV+ ABV,
pac56	x	x	x	x									x	x																	x		x	x	x	x	x	x	x	x	x	x	x	x			x	x	x	x					x	x	x	x	x	x	x					x	x	x		x							IDV, RTV+SQV, RTV+NFV	AZT+ddI, 3TC+d4T, d4T+NVP

Table S3. Mutations (Pre- & Post- ATI) by Codon and ARV Drugs Used by Patients Prior to ATI. "x" indicates that a mutation was present. The drug names are indicated in Table S1.
