# Supplementary material for: Fitness Cost of Antiretroviral Drug Resistance Mutations on the pol Gene during Analytical Antiretroviral Treatment Interruption among Individuals Experiencing Virological Failure
Source: Pathogens. 2021 Nov 3;10(11):1425. doi: 10.3390/pathogens10111425 (PMC8622617; doi:10.3390/pathogens10111425)
Supplement: Supplementary file 1 [file pathogens-10-01425-s001.zip › pathogens-1266514-supple/S4_table_pol.pdf]

Study – 1st Author	Year	Journal	Participants
Miller	2000	AIDS	48
Katlama	2004	AIDS	68
Deveraux	1999	AIDS	25
Izopet	2000	AIDS	38
Deeks	2003	AIDS	24
Neumann	1999	AIDS	10
Papasavvas	2003	AIDS	11
Harrigan	1999	AIDS	6
Garcia	1999	AIDS	8
Deeks	2001	NEJM	16
Dybul	2001	PNAS	10
Aranowich	2003	AIDS	36
Maggiolo	2004	AIDS	69

Journal Names

Abbreviation	Full Journal Name
AIDS	AIDS: A Journal of the International AIDS Society
NEJM	New England Journal of Medicine
PNAS	Proceedings of the National Academy of Sciences
