# Supplementary figures and images for: Fitness Cost of Antiretroviral Drug Resistance Mutations on the pol Gene during Analytical Antiretroviral Treatment Interruption among Individuals Experiencing Virological Failure
Source: Pathogens. 2021 Nov 3;10(11):1425. doi: 10.3390/pathogens10111425 (PMC8622617; doi:10.3390/pathogens10111425)

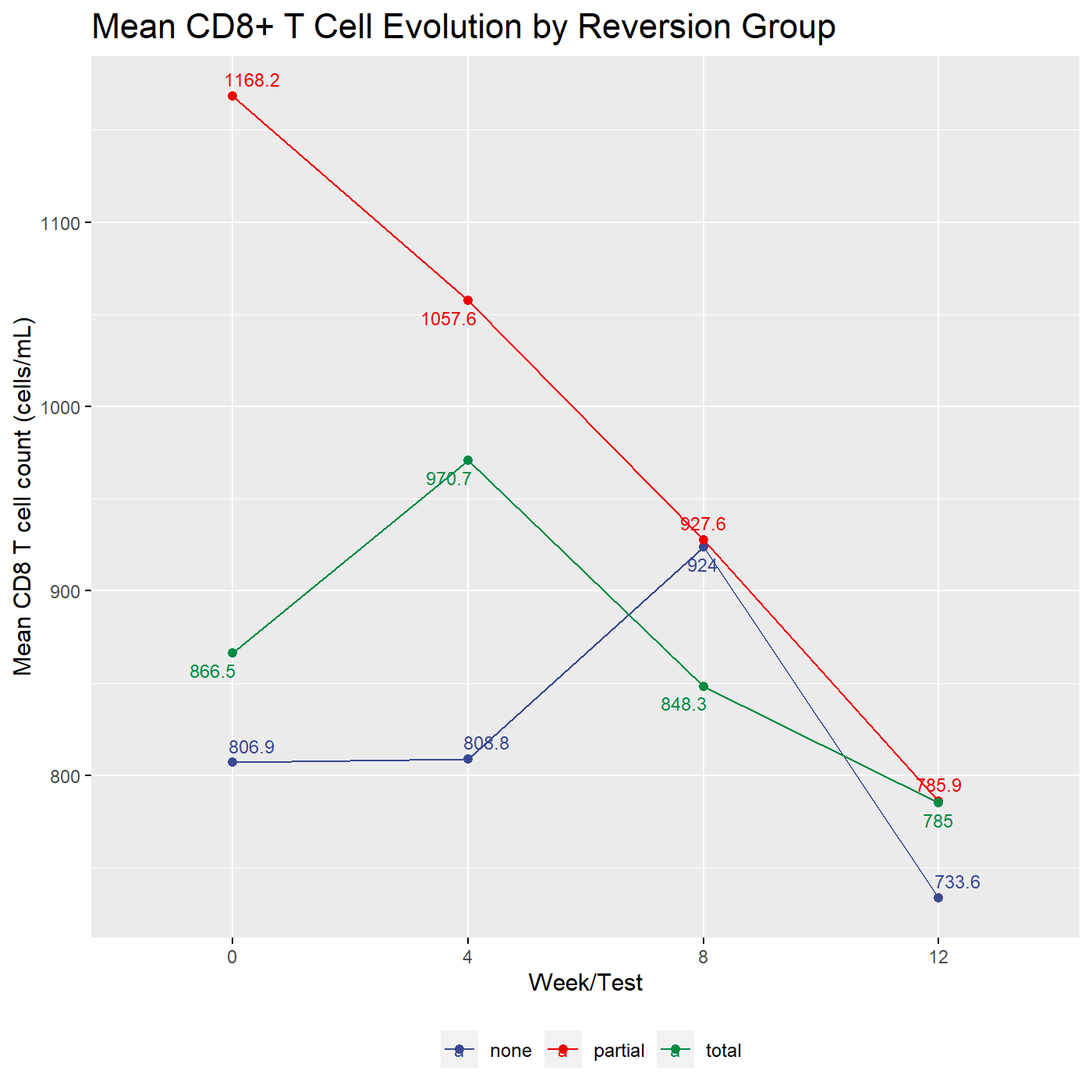

Supplement: Supplementary file 1 [file pathogens-10-01425-s001.zip › pathogens-1266514-supple/fig_s1_pol_oct.tiff]

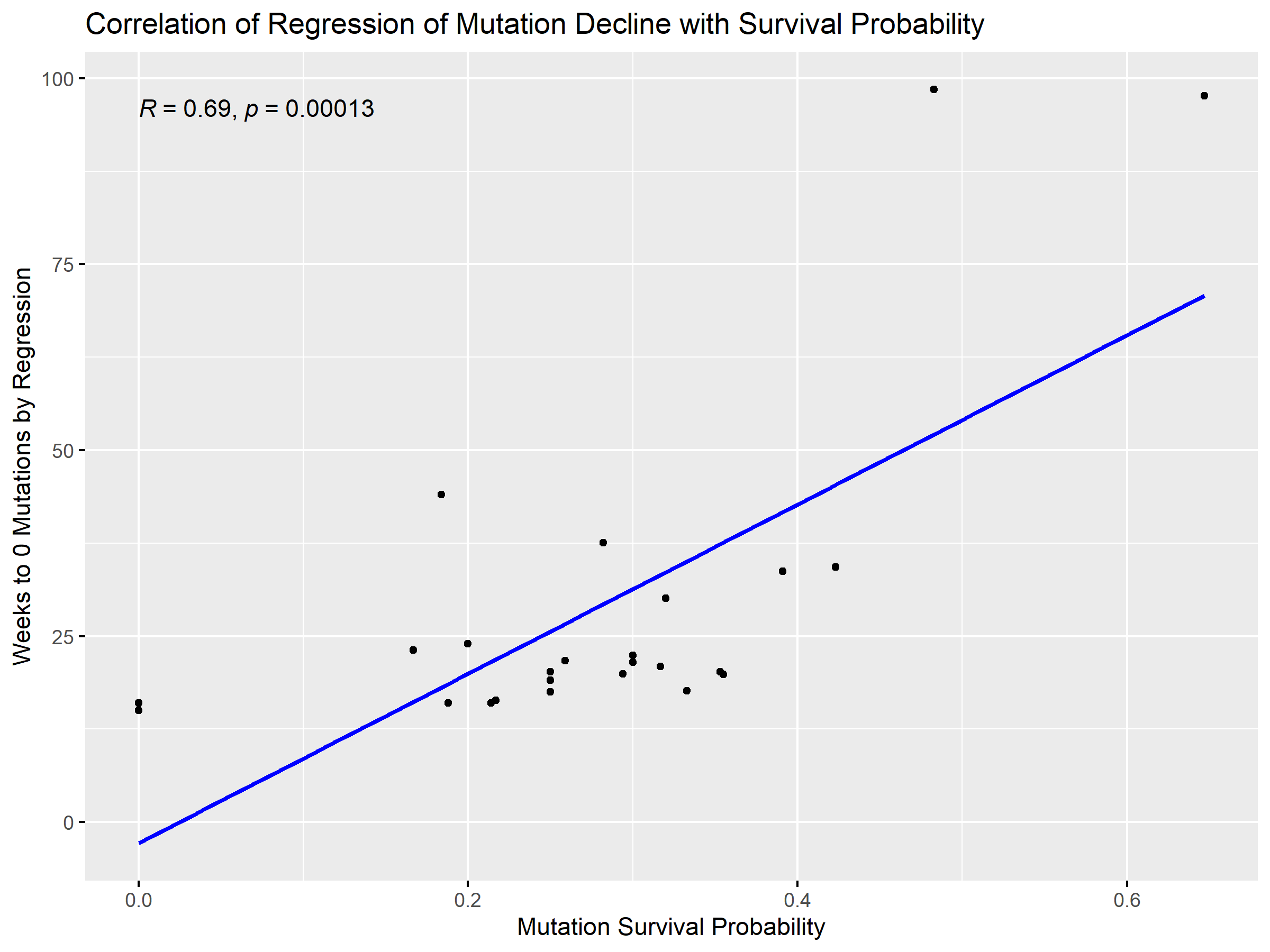

Supplement: Supplementary file 1 [file pathogens-10-01425-s001.zip › pathogens-1266514-supple/fig_S2_pol.tiff]
